# Vaccine Information Seeking Behavior Among Pregnant Women in Khartoum State, Sudan: A Hospital-Based Cross-Sectional Study

**DOI:** 10.3389/fpubh.2021.586333

**Published:** 2021-06-25

**Authors:** Majdi M. Sabahelzain, Zienab A. Ibrahim, Sahar A. B. Hamad, Gary Finnegan

**Affiliations:** ^1^Department of Public Health, School of Health Sciences, Ahfad University for Women, Omdurman, Sudan; ^2^Tat3im Initiative (Tat3im=immunization), School of Health Sciences, Ahfad University for Women, Omdurman, Sudan; ^3^Federal Ministry of Health, Khartoum, Sudan; ^4^School of Medicine, Ahfad University for Women, Omdurman, Sudan; ^5^Vaccines Today, Brussels, Belgium

**Keywords:** vaccine information, seeking behavior, Khartoum, Sudan, pregnant women, hospitals, vaccine confidence

## Abstract

**Objective:** This study aims to explore vaccine information-seeking behavior and its determinants among pregnant women in Khartoum state, Sudan. The findings from this study will be used to inform further development of policies and interventions in Sudan to increase vaccine acceptance and demand.

**Methods:** A hospital-based cross-sectional study was conducted in two public hospitals, Omdurman maternity and AL-Saudi hospitals in Omdurman, Khartoum state, from February to April 2020.

**Results:** We interviewed 350 pregnant women in the two hospitals. Our findings showed that one-third of pregnant women (35.7%) searched for information about vaccines. The vast majority searched for this information before pregnancy and during pregnancy (34.4 and 59.2%, respectively). They primarily searched for topics related to vaccine schedules and vaccine side effects (28.8% for each). The main sources of vaccine-related information consumed by pregnant women were healthcare professionals, particularly doctors (40%), and the internet (20.8%). Findings showed that a high level of education was associated with a greater likelihood of searching for additional vaccine information. Moreover, those who perceived their family to have a high income were more likely to search for information. Additionally, pregnant women with low confidence in vaccines were more likely to be involved in searching for additional vaccine information. This highlights the need for high-quality, easily accessible information that addresses their needs.

**Conclusion:** Our findings showed that confidence in vaccine influences seeking for relevant information. We recommend the development of client-centered communication interventions to help increasing vaccine confidence and consequently vaccine acceptance and demand.

## Introduction

Health awareness is the first step in the process of promoting healthy behavior (1). Health information can be actively sought or passively received from various sources. Healthcare providers were found as the most trusted source of reliable information about vaccines (2–4). Studies from high-income countries showed that most people seek information about vaccines from the internet (5–7). However, searching on the internet has been shown to bring web users to low-quality anti-vaccine websites that disseminate misinformation, myths, and conspiracy theories about vaccines (5).

Therefore, the World Health Organization established the Vaccine Safety Net (VSN) in 2003 as an international network of websites that delivers reliable information on vaccine safety (8). Vaccine information-seeking behavior has been studied extensively in high-income countries. However, there is a dearth of research from low- and middle-income countries (LMIC) including Sudan. The scarcity of official trusted sources for vaccine information encouraged academic institutions to initiate web-based interventions such as the Tat3im Initiative website (Tat3im means immunization in the Arabic language). It is a Sudanese website which is a member of the WHO-led project VSN (9) and aims to increase the awareness and literacy of vaccines, immunization, and vaccine-preventable diseases.

Therefore, this study aims to explore vaccine information-seeking behavior and its determinants among pregnant women in Omdurman Maternity and Al-Saudi hospitals in Khartoum state, Sudan. The findings from this study will be used to inform further development of policies and interventions in Sudan to increase vaccine acceptance and demand.

## Methods

### Study Design and Setting

A hospital-based cross-sectional study was conducted in two hospitals, Omdurman maternity and AL-Saudi hospitals in Omdurman, Khartoum state, from February to April 2020. These two hospitals were selected because both are specialized in providing obstetric and gynecological services to public. Therefore, they are well-placed to meet the study's goal of engaging with pregnant women.

### Population and Sampling Method

The study population consists of Sudanese pregnant women aged 15–49 years who attended either of the two hospitals at the time of the study. The estimated sample size was 315. However, to cover for possible dropouts due to missing information on crucial questions, a total of 350 participants were recruited for the study.

The sample size was computed using the following formula (10):

$$\begin{array}{l} \left[n\;=\;\frac{z^{2}pq}{d^{2}}\;\right] \end{array}$$

Where

*n* = sample size
*z* = (1 −α), is the z-score corresponding to a 95% confidence interval and was computed as 1.96.
*p* = 0.288 which is the probability/percentage of women who received at least two doses during the last pregnancy in Khartoum state based on the 2014 MICS (11).
*q* = (1 − p) = 0.712
*d* = desired margin of error or 0.05.

Unpublished data from the two hospitals showed that the minimum numbers of patients' visits per month are 1,500 and 1,100 for the Omdurman Maternity and Al-Saudi hospitals, respectively. Accordingly, we selected 200 participants from Omdurman Maternity and 150 participants from Al-Saudi hospitals with respect to the size proportions in terms of patients' visits. Systematic random sampling was used to enroll pregnant women in this study.

### Study Instrument and Study Variables

Data was collected using a pretested questionnaire. The interviews were conducted in the Arabic language with every eighth woman who attended the public clinics until the estimated sample size was completed from each hospital. Data was collected using the pretested questionnaires obtained from pregnant women after their consultation with doctors. The dependent variable was vaccine information-seeking behavior, which was measured by asking the participants whether they have ever searched for additional vaccine information rather than information that is provided by vaccinators. The independent variables included sociodemographic data, testing the general knowledge about tetanus and tetanus toxoid vaccine (10-item scale), confidence in vaccine by using the Vaccine Confidence Index (VCI) which contains four statements about vaccine importance, safety, efficacy, and its compatibility to the religious beliefs (12), number of pregnancies, and planning of the current pregnancies in addition to tetanus vaccination status of the pregnant women.

### Statistical Analysis

Data analysis was performed using Statistical Package for Social Sciences (SPSS) software (Version 24). The chi-square, Fisher's exact (when the count is <5 in a cell), or Mann–Whitney U (for Knowledge and VCI scores) test was conducted to assess the factors associated with vaccine information-seeking behavior among pregnant women. Variables that were significantly associated with the primary dependent variable were included in a multivariable logistic regression model to identify the predictors of vaccine information-seeking behavior among pregnant women. A *p*-value of < 0.05 was considered statistically significant.

### Ethical Consideration

Ethical approval in a written format was obtained from the Ahfad University for Women research ethics committee. Permissions to enter the hospitals were obtained from the Khartoum State Ministry of Health and the hospitals' general managers.

## Results

### Descriptive Statistics

Of the 350 pregnant women, 57.1% attended the Omdurman Maternity hospital. The mean age of the pregnant women who participated in the study was 28.41 (SD = 6.28). About half of the respondents (47%) completed university. The majority (89.7%) of the respondents self-reported that they had medium income level. Nearly less than half of the respondents (44%) mentioned that they experienced previously one to two pregnancies, while the rest mentioned that they either had three to four or five and more pregnancies (28% for each). More than half of the respondents (57.7%) reported that the current pregnancy was unplanned. About half of the respondents vaccinated partially with the tetanus vaccines (<2 doses). Additionally, the chi-square test (or Fisher's exact tests when necessary) was performed to assess the association between searching for additional information about vaccination (vaccine information seeing behavior) and the sociodemographic factors. We found that vaccine information-seeking behavior was highly associated with the education level of the pregnant women, as mothers with secondary were more likely to search for additional information followed by those who reported University education (*p*-value < 0.001). Vaccine information-seeking behavior increased with the increase in income level of pregnant women's household (self-ranking income level) (*p*-value = 0.016). Furthermore, there is a difference in vaccine information-seeking behavior between those who attended the Omdurman Maternity and Al-Saudi hospitals, as those who attended the Al-Saudi hospital were more likely to search for additional information about the vaccine (*p*-value = 0.019).

### Information-Seeking Behavior

**Figure 1:**
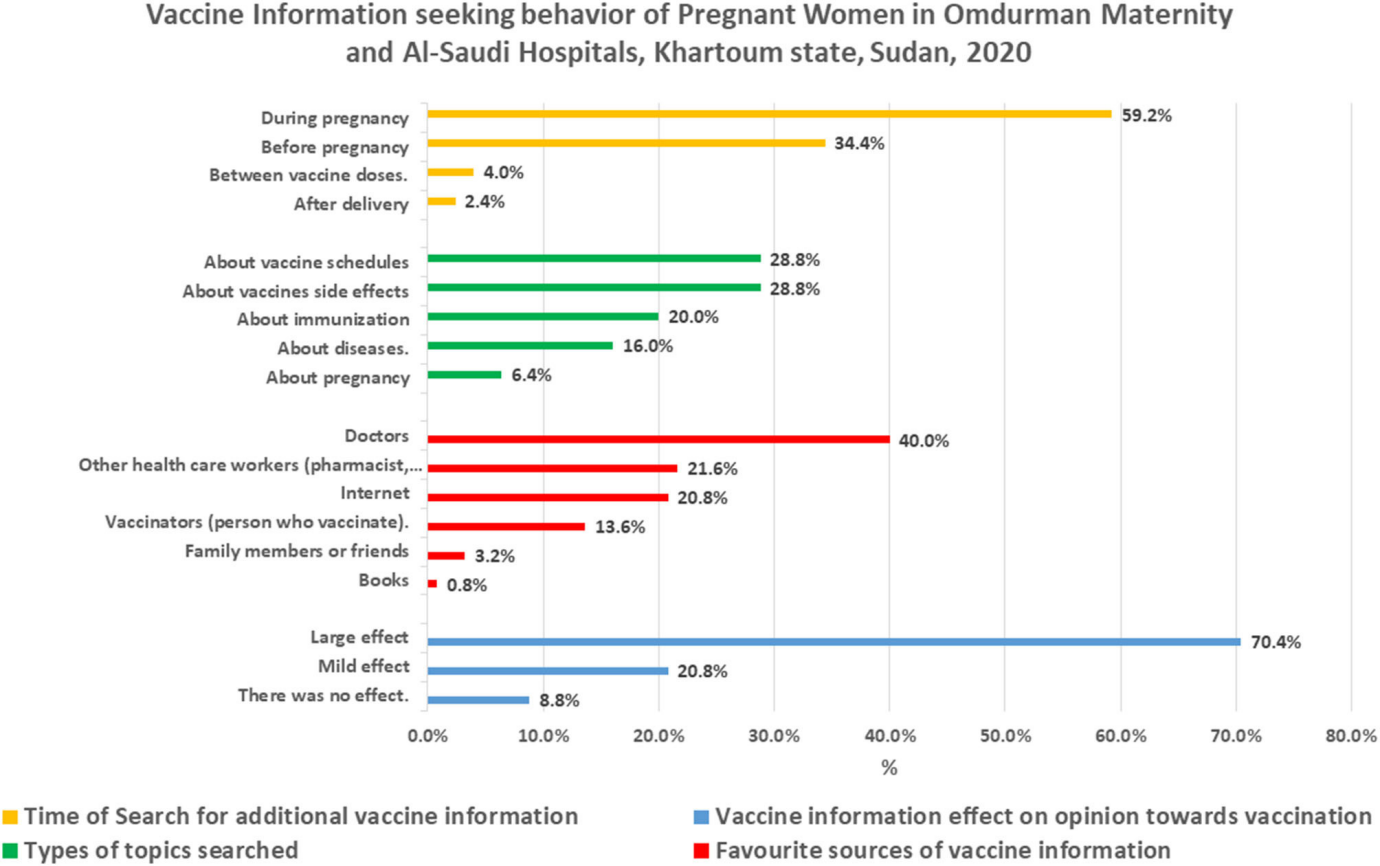
Vaccine information-seeking behaviors of pregnant women (*N* = 350).

As shown in Table 1, one-third of the respondents (35.7%) reported that they have searched for additional information about vaccination (vaccines/diseases). Most of them (59.2%) searched for this information during pregnancy, followed by a third (34.4%) who searched before pregnancy (Figure 1).

**Table 1:** Sociodemographic characteristics and associations with vaccine information-seeking behavior (*N* = 350).

		**Searching for additional information about vaccination (vaccines/diseases)**			
		**No = 225 (64.3) *N* (%)**	**Yes = 125 (35.7) *N* (%)**	**Total *N* (%)**	***p-*value**
Hospital	Omdurman maternity	139 (69.5)	61 (30.5)	200 (57.1)	0.019^*^
	AL-Saudi	86 (57.3)	64 (42.7)	150 (42.9)	
Education level	Not educated	6 (100.0)	0 (0.0)	6 (1.6)	<0.001^*^^a^
	Primary	88 (83.0)	18 (17.0)	106 (30.3)	
	Secondary	30 (41.7)	42 (58.3)	72 (20.6)	
	University	101 (60.8)	65 (39.2)	166 (47.4)	
Income level (self-ranking)	High	7 (50.0)	7 (50.0)	14 (4.0)	0.016^*^
	Medium	198 (63.1)	116 (36.9)	314 (89.7)	
	Low	20 (90.9)	2 (9.1)	22 (6.3)	
Number of pregnancies	1–2	100 (64.9)	54 (35.1)	154 (44.0)	0.882
	3–4	61 (62.2)	37 (37.8)	98 (28.0)	
	5 and more	64 (65.3)	34 (34.7)	98 (28.0)	
Planning of the current pregnancy	Unplanned	132 (65.3)	70 (34.7)	202 (57.7)	0.628
	Planned	93 (62.8)	55 (37.2)	148 (42.3)	
Tetanus vaccination status	Not vaccinated	18 (75.0)	6 (25.0)	24 (6.9)	0.198
	Partially vaccinated	97 (59.9)	65 (40.1)	162 (46.3)	
	Fully vaccinated	82 (64.6)	45 (35.4)	127 (36.3)	
	Don't know	28 (25.7)	9 (24.3)	37 (10.5)	

* *Statistically significant (p-value < 0.05)*.

a *Fisher's exact test*.

The most common topics that the respondents searched for were vaccine side effects and vaccine schedules (28.8% for each). The preferred information sources used most frequently were their doctor (40%), other healthcare workers (such as pharmacists, nurses) (21.6%), and the Internet (20.8%). Among the respondents who used the internet, almost two-thirds (65.2%) used the Google search engine, followed by those who searched health services' websites (13%) and Facebook (8.7%). More than two-thirds (70.4%) of the respondents, who search for additional information, reported that the information they found had a large effect on their opinion toward vaccination.

### Predictors of Vaccine Information-Seeking Behavior of Pregnant Women

To get insights on the predictors of vaccine information-seeking behavior among pregnant women, a multivariable logistic regression model was run including all variables that were statistically significant (Table 1), as well as two variables which were analyzed using the Mann–Whitney *U*-test (the VCI, *U* = 12, 421.5, *p* = 0.055, and knowledge about tetanus and its vaccine, *U* = 11, 954.5, *p* = 0.020).

The logistic regression analysis results are summarized in Table 2. Compared to pregnant women with University education, those whose formal education did not proceed beyond secondary school were twice as likely to search for additional information about vaccination (odds ratio (OR) = 2.00, *p* < 0.05). However, those with primary education only were less likely to search for additional information (odds ratio (OR) = 0.32, *p* < 0.05). With regard to perceived wealth, pregnant women who reported that they belong to a family with high income level were almost nine times more likely to search for additional information about vaccination than those who self-ranked their income level as low (OR = 8.88, *p* < 0.05). Moreover, the VCI is significantly predictive of whether pregnant women will search for additional information about vaccination. As the VCI score increases, the odds of a pregnant woman searching for additional information decreases (OR = 0.56, *p* < 0.05; see Table 2).

**Table 2:** Predictors of vaccine information seeking behavior among pregnant women.

**Predictors**	**OR_**adj**_**	**95% C.I of OR**		***p*-value**
		**Lower**	**Upper**	
**Hospital**				
Omdurman maternity	0.721	0.443	1.174	0.189
AL-Saudi (ref)	–	–	–	–
**Education level**				
Not educated	0.000	0.000		0.999
Primary	0.315	0.169	0.590	<0.001^*^
Secondary	2.009	1.113	3.628	0.021^*^
University (ref)	–	–	–	–
**Family income (self-ranking)**				
High	8.884	1.365	57.835	0.022^*^
Medium	3.631	0.777	16.959	0.101
Low (ref)	–	–	–	–
Vaccine confidence index	0.562	0.331	0.956	0.033^*^
Average knowledge about tetanus	0.819	0.481	1.396	0.464

* *Statistically significant (p < 0.05)*.

*OR_adj_, adjusted odds ratio; Ref, reference category*.

## Discussion

This study aimed to understand vaccine information-seeking behavior and its predictors among pregnant women in two hospitals specialized in maternity care. Our findings showed that one-third of pregnant women (35.7%) searched for information about vaccines. The vast majority searched for this information when they expected pregnancy and became pregnant (34.4 and 59.2%, respectively). They primarily searched for topics related to vaccine schedules and vaccine side effects (28.8% for each). Similar results from Germany showed that 35% of pregnant women reported a need for further information, mainly for vaccine side effects, whether for themselves or for their unborn child (13). These key moments and topics offer windows to address pregnant women with intensive campaigns and programmatic interventions.

The study findings showed that pregnant women actively sought advice about vaccines from healthcare professionals, particularly doctors (40%). About one-fifth of the pregnant women (20.8%) reported that they look for additional information about vaccines online. Many studies support our findings that people highly trust healthcare professionals as a reliable source of vaccine information (2).

Although the importance of the internet in searching for vaccine information is still low in Sudan, web users who search for such information are at risk of experiencing low-quality anti-vaccine websites that possibly propagate myths and conspiracy theories (5, 14).

Our study findings showed that pregnant women who attended secondary school have twice the likelihood to search for additional vaccine information compared to those who completed university. Moreover, those who perceived themselves to have high levels of family income were more likely to search for such information. These findings suggest that there is inequality regarding vaccine information-seeking behavior. Therefore, interventions aimed at addressing broader issues like access to education and information may have a positive impact on this healthy behavior.

Interestingly, we found that pregnant women with low confidence toward vaccine were more likely to be involved in searching for additional vaccine information. This finding is supported by different studies that suggest that low satisfaction and lack of trust in vaccine information provided by the healthcare professionals might motivate people to search for additional information to satisfy their needs (4, 15–17). As vaccine hesitancy is prevalent globally, we suggest that vaccine information-seeking behavior might be associated with the hesitancy toward vaccination (18).

Within the current COVID-19 pandemic context, different issues related to vaccination especially the infodemic (the epidemic of misinformation), and the global disruption of the existing routine vaccination especially in the LMICs that accompanied COVID-19, drew great attention by the global health bodies including WHO and UNICEF during the COVID-19 pandemic early this year (19, 20). Therefore, our study's findings are needed and provided timely relevant knowledge that can be used in developing policies and interventions to reduce the impacts of the issues that accompanies the COVID-19 pandemic in Sudan.

We conclude that our study findings showed that confidence in vaccine influences seeking for related information. We recommend development of client-centered communication interventions to help increase vaccine confidence and consequently vaccine acceptance and demand. As the pregnant women who had secondary school education (i.e., but not University education) were the most likely to search for information, we suggest that further research is needed to explore these matters.

## Limitation

The result of this study should be interpreted given the context of the study, as the respondents were recruited only from two hospitals in Khartoum state which may not represent the whole population of pregnant women in Sudan. The interviews were conducted during their rest time after finishing their consultation with doctors, which was not convenient for some pregnant women. Additionally, about half of the pregnant women (47.4%) reported the completion of their University education. This figure is higher than the average of higher education attendance rates for the females (about 15 and 30% at the national and Khartoum state levels, respectively). However, given the purpose of the study, we do not expect these limitations in the study to have any significant impact on our findings.

## Data Availability Statement

The raw data supporting the conclusions of this article will be made available by the authors, without undue reservation.

## Ethics Statement

The studies involving human participants were reviewed and approved by Ahfad University Review Board and Ethical Committee at the ministry of Health. The patients/participants provided their written informed consent to participate in this study.

## Author Contributions

The study was initially designed by MS and ZI then discussed by SH and GF. ZI collected the data, and MS conducted the data analysis. The results were discussed collectively. MS and ZI drafted the paper. All authors contributed to the article and approved the submitted version.

## Conflict of Interest

GF is employed by the Vaccines Today which is funded by Vaccines Europe. The remaining authors declare that the research was conducted in the absence of any commercial or financial relationships that could be construed as a potential conflict of interest.
